# Editorial: Neuroimaging Biomarkers and Cognition in Alzheimer's Disease Spectrum

**DOI:** 10.3389/fnagi.2022.848719

**Published:** 2022-02-25

**Authors:** Jiu Chen, Siyu Wang, Rong Chen, Yong Liu

**Affiliations:** ^1^Institute of Neuropsychiatry, The Affiliated Brain Hospital, Fourth Clinical College, Nanjing Medical University, Nanjing, China; ^2^Fourth Clinical College, Nanjing Medical University, Nanjing, China; ^3^Department of Diagnostic Radiology and Nuclear Medicine, University of Maryland School of Medicine, Baltimore, MD, United States; ^4^School of Artificial Intelligence, Beijing University of Posts and Telecommunications, Beijing, China

**Keywords:** Alzheimer's disease spectrum, subjective cognitive decline, mild cognitive impairment, neuroimaging, cognition

Subjective cognitive decline (SCD) and mild cognitive impairment (MCI) are well recognized to be at high risk of converting to Alzheimer's disease (AD) and act as a clinical continuum of the AD spectrum. Neuroimaging is applied as a tool to figure out both anatomical and functional alterations in the AD spectrum and may further reveal the pathophysiologic mechanism of AD. This topic aimed to recruit worldwide articles focusing on neuroimaging biomarkers and cognition in the AD spectrum. A total of 30 contributions from 168 different authors have been included as of January 2022.

## Structural and Metabolic Alterations Correlated With Cognition in the AD Spectrum

Voxel-based morphometry (VBM) is a useful tool for detecting structural alterations in the preclinical AD spectrum. Zhang J. et al. assessed 31 VBM studies in amnestic MCI (aMCI) patients, discovering the highly robust deterioration in the left amygdala and right hippocampus. This indicates that specific gray matter (GM) atrophy may serve as a potential biomarker for early AD diagnoses. GM atrophy in the hippocampus and its subfield (HippSub) has long been recognized as typical lesions of AD. Several articles in our Research Topic focus on alterations in hippocampal volume. For example, Hansen et al. discovered alterations in HippSub volume in AD, but not in bipolar disorder (BD), or in major depressive disorder (MDD). This study reinforced the notion of different neural mechanisms in hippocampal degeneration. Except for HippSub volumes, atrophy in other brain regions has been observed to be correlated with cognitive dysfunction. Chen Q. et al. discovered reduced basal forebrain (BF) volume, especially in the Ch4p subfield in SCD patients compared with healthy controls, which was further associated with spatial disorientation. This indicates a structural basis for allocentric disorientation independent of hippocampal atrophy in SCD patients. To note, there is an increasing number of studies focusing on subdivided brain regions. We may expect that precisely subdividing regions could assist in improving the accuracy of locating preclinical AD lesions and further exploring the essence of disease changes.

White matter (WM) fiber bundles communicate with various brain regions and serve as important structural components of the brain. AD progression leads to potential damage to WM. One typical pathological change is white matter hyperintensities (WMHs). WMH reflects demyelination, which is a deterioration of neural pathways caused by decreasing blood flow and/or disease. WMHs have been widely observed in dementia such as AD. At the same time, diffusion tensor imaging (DTI) is used for detecting water molecule diffusion and is sensitive in white matter atrophy detection. Fractional anisotropy (FA), mean diffusivity (MD), and relative anisotropy (RA) are recognized as common DTI metrics. Diaz-Galvan et al. assessed cerebrovascular disease by applying WM signal abnormalities (WMSA) and MD. They further combined correlation, multiple regression, and mediation analyses to investigate the association between depressive symptomatology, cerebrovascular disease, and SCD. Likewise, several contributions of WM pay special attention to lesions in specific brain regions. Srisaikaew et al. focused on disruption of fornix integrity and fiber length in non-amnestic MCI (naMCI) compared to healthy controls, and its association with cognition. Based on their study results, the authors suggested that fornix fiber tract length played a crucial role in sustaining executive function in naMCI patients. To summarize, various studies focus on WM and GM separately. Still, since GM and WM together constitute the major part of the cerebrum, the internal relationship of atrophy between GM and WM is worth pondering, which may reveal the in-depth deteriorative and compensatory mechanism of the AD spectrum.

As for micro-alterations, fluorodeoxyglucose-B-positron emission tomography (FDG-PET) is utilized to reveal glucose metabolism in patients of the early AD spectrum. By GM volume analyses and glucose metabolism analyses, Lee S.-Y. et al. announced that tinnitus may lead to abnormal metabolism and altered cerebral architecture in MCI patients, providing insights into the combination of micro (metabolism) and macro (cerebellar structure) alterations to reveal AD pathology. Aβ and tau pathology are well established as AD typical biomarkers. By conducting partial correlation analyses under different amyloid statuses, Ge et al. calculated the relationship between tauopathy/volume of the hippocampal subfields and assessment scores. A significant decrease in hippocampal volume and increase in tau deposition of hippocampal subfields were observed in the Aβ-positive group compared to the negative one, indicating the feasibility of applying neuroimaging methods to explore traditional biomarkers.

## Functional Alterations, Network Connectivity, and Entropy Mapping Correlated With Cognition in the AD Spectrum

Resting-state functional magnetic resonance imaging (rs-fMRI) is widely applied for AD early detection by means of functional connectivity (FC). Wang S. et al. applied data from the Nanjing Brain Hospital-Alzheimer's Disease Spectrum Neuroimaging Project (NBH-ADsnp) database (Dr. Jiu Chen serves as the principal investigator of NBH-ADsnp) to conduct FC in insular subnetworks for SCD and aMCI classification. Amplitude of low-frequency fluctuations (ALFF) can be utilized for measuring low-frequency oscillations of the blood-oxygen-level-dependent (BOLD) signal and localize altered spontaneous brain activities. Zhang X. et al. looked into previous studies on ALFF and fractional ALFF (fALFF) in amnestic and vascular MCI patients, suggesting the possibility of applying ALFF/fALFF for distinguishment. Except for FC and ALFF, other assessments such as regional homogeneity (ReHo), etc. can reflect neuronal activities.

Minor alterations pile up and eventually lead to the dysfunction of network connectivity. Many contributions in our Research Topic focus on brain networks. Sheng et al. conducted graph theory analysis to explore altered GM network metrics among healthy controls, MCI patients, and AD patients. Their study provided insight into the association between cognitive impairment and brain structural network compensation. To reveal executive function (EF) alterations, Liu et al. calculated fALFF and FC in an executive control network (ECN) and examined the relationship between altered fALFF or FC and EF composite score, uncovering the convergence and divergence in the MCI-high EF group and MCI-low EF group. Moreover, Lee P.-L. et al. suggested that the posterior cingulate cortex (PCC)-synchronized degeneration network (SDN) is spatially correlated with patterns of the GM atrophy rate, which is in better association with the AD spectrum than hippocampus-SDN. These contributions uncovered the deterioration and compensation of preclinical AD correlated to cognitions under diverse brain networks, which may also assist in revealing disease mechanism and progression.

Progressive brain deterioration leads to increasing brain entropy (BEN). In order to characterize BEN in AD and test the inverse-U-shape BEN model, Wang Z. et al. compared BEN between AD and normal aging, and further correlated BEN with age, education, etc. Abnormal decreasing BEN was discovered in association with severe cognitive impairment and daily function disability in an AD group, indicating an inverse-U trajectory of BEN evolution when normal aging progresses into AD dementia.

## Potential Classification Tools for the AD Spectrum

Optimizing classification tools for the early AD spectrum has always been a pursuit for researchers in this field. Research is conducted mainly by applying machine learning, pattern recognition, logistic regression, and deep learning strategy. For example, by integrating altered rCBF, ALFF, and ReHo, Zhang Q. et al. established a support-vector classifier model of machine learning to classify patients of the AD spectrum from HC. Except for applying various biomarkers, Wang S.-H. et al. proposed an Alzheimer's Disease VGG-Inspired Attention Network (ADVIAN) to better identify AD. Some researchers also chose to optimize the existing models. Du et al. suggested that anisotropy of anomalous diffusion improved the accuracy of classifying AD in a novel fractional motion model. These studies cast light on the improved strategy of AD diagnoses. Still, more effort is needed for establishing AD diagnostic models based on the proper combination of stable biomarkers, which are accurate, convenient, and economical enough for clinical use.

## Potential Prevention Strategy for the AD Spectrum

Two other contributions focusing on intervention are included in our Research Topic. To measure the effect of aerobic exercises, Yu et al. examined hippocampal volume, temporal meta-regions of interest (ROI) cortical thickness, WMH volume, and network failure quotient (NFQ). They further performed correlation analyses between 6- and 12-month changes of MRI biomarkers and the AD Assessment Scale-Cognition (ADAS-Cog). The results revealed that hippocampal volume and temporal meta-ROI cortical thickness are slightly reduced only during the intervention period. By applying probabilistic tractography and voxel-based morphometry, Kim G.-W. et al. discovered that no significant changes in thalamo-cortical WM connectivity, cortical thickness, or GM volume exist between MCI patients with/without donepezil treatment. A reliable intervention strategy is urgently needed for slowing down the procession of AD. Thus, carrying out relevant longitudinal research is a necessity. Through structural and functional alterations and correspondent clinical symptoms, these two studies assessed the treatment effect of aerobic exercises and donepezil for preclinical stages of AD. This suggests that potential neuroimaging biomarkers can be further applied to evaluate the impact of diet, lifestyle intervention, and medication on AD progression. At the same time, the combination of multimodal interventions may have superimposed therapeutic effects, which is also worthy of researchers' attention.

## Author Contributions

JC, RC, and YL designed the topic. JC and SW prepared the manuscript. All authors contributed to the article and approved the submitted version.

## Funding

This study was supported by the National Natural Science Foundation of China (No. 81701675), the Key Project supported by Medical Science and Technology Development Foundation, Nanjing Department of Health (No. JQX18005), and the Key Research and Development Plan (Social Development) Project of Jiangsu Province (No. BE2018608).

## Conflict of Interest

The authors declare that the research was conducted in the absence of any commercial or financial relationships that could be construed as a potential conflict of interest.

## Publisher's Note

All claims expressed in this article are solely those of the authors and do not necessarily represent those of their affiliated organizations, or those of the publisher, the editors and the reviewers. Any product that may be evaluated in this article, or claim that may be made by its manufacturer, is not guaranteed or endorsed by the publisher.

